# Shifting Power in Practice: The Importance of Contextual and Experiential Evidence in Guiding MCH Decision Making

**DOI:** 10.1007/s10995-022-03457-8

**Published:** 2022-07-19

**Authors:** Laura Powis, Grace Guerrero Ramirez, Lynda Krisowaty, Benjamin Kaufman

**Affiliations:** 1 Association of Maternal & Child Health Programs (AMCHP), 1825 K Street, Suite 250, 20006 Washington, DC USA; 2 Mathematica, Oakland, CA USA

**Keywords:** Evidence, MCH workforce development, Community-defined evidence, Contextual evidence, Experiential evidence, Power shifting, Title V

## Abstract

**Background:**

Evidence is central to all maternal and child health (MCH) decision-making processes, continuously interacting with and influencing our work. There is a growing emphasis in MCH on using evidence-based approaches when addressing public health challenges, but the field lacks a unified understanding of what constitutes evidence. MCH must operate from an expansive understanding of evidence that centers community voice and acknowledges the role of evidence prioritization in achieving equitable population-level outcomes.

**Call to Action:**

What we consider valid evidence has immense implications for MCH practice, including whose work is deemed worthy of funding and replication. The authors advocate for shifting the field’s evidence paradigm from being primarily focused on research findings to also recognize the importance of community-rooted evidence. Contextual and experiential evidence, alongside research evidence, should be considered for two purposes: (1) to support the effectiveness of a given practice generally, and (2) to support that the practice will work in specific contexts.

Putting this shift into practice requires explicit power shifting – the MCH workforce must cede power to those who have been historically barred from participating in and guiding research. To facilitate this, MCH professionals must build skills in communication, equitable leadership, and change management.

**Conclusion:**

The MCH workforce should position communities to set their own priorities and define, develop, and disseminate evidence representative of their priorities. Evidence co-creation is key to establishing and sustaining transformative relationships between community members and Title V programs, shifting power structures to build upon existing community leadership and assets.

## Significance

Evidence is key to maternal and child health (MCH) decision-making and programming. Although what the field considers evidence is evolving to recognize community preferences and insights, MCH programming is prioritized towards research-based evidence that often excludes its intended beneficiaries.

We propose a paradigm shift in how we consider evidence to center the values, needs, and expertise of impacted communities and those with lived experience, and posit that shifting power is essential to putting this approach into action. Evidence co-creation is an essential pathway to building and sustaining trust and is key to advance racial and other forms of equity.

## Introduction

Imagine yourself in this hypothetical, but hopefully resonant scenario: After identifying priorities through your jurisdiction’s five-year Needs Assessment, you’ve been assigned by your supervisor to develop, seek sustainable funding for, and implement a practice aimed at achieving more equitable outcomes within your maternal and child health (MCH) domain of expertise. You scan funding sources and target a federal notice of funding opportunity to increase service delivery capacity within jurisdictions. You also identify a request for proposals from a private foundation whose mission includes addressing health needs in your geographic region. Responding to these opportunities requires your team to include “evidence-based” approaches in your proposal. But what does “evidence-based” mean? What comes to mind when you see the word “evidence” and where does your understanding of this term come from?

A final, more encompassing question: How does what we consider “evidence” influence the practices we design, implement, or fund?

## Historical Context

### What is evidence?

MCH commonly uses phrases like “evidence-based” to label effective practices, but there is no clear definition in the field as to what constitutes “evidence” (Puddy & Wilkins, 2011; APA, 2005; Canadian Health Services Research Foundation 2004; Lomas et al., 2005; Canadian Health Services Research Foundation, 2006; Substance Abuse and Mental Health Services Administration, 2008). At a basic level, evidence is proof or demonstration that something (we use “practice” or “practices” in this commentary) works. For some, this term might immediately call to mind laboratory experiments or academic studies. Others might consider quantitative or qualitative data, or ideas shared by members of the communities that the MCH field intends to benefit. When state/jurisdictional Title V staff were asked this question during the National MCH Workforce Development Center’s 2021 and 2022 Learning Institutes, some of the most common answers were: “peer reviewed journals,” “science/research,” and “documentation of proof.” The variety of answers underscores the array of mental models we hold about evidence.

Given the increasing emphasis placed on using evidence-based approaches when addressing public health challenges, it is critical that we examine this topic more closely to ensure that MCH operates from an expansive and unified definition of evidence, and that the MCH workforce acknowledges the role of evidence prioritization in achieving equitable population-level outcomes (Puddy & Wilkins, 2011; Substance Abuse and Mental Health Services Administration, 2008).

### Where Do These Mental Models Come From?

To gain insight into where our understanding of evidence comes from, we must first look to evidence-based medicine (EBM) – the “conscientious, explicit, judicious, and reasonable use of modern, best evidence in making decisions about the care of individual patients” (Sackett et al., 1996). Over the past two decades, EBM has increasingly been accepted as the gold standard for decision making, with randomized control trials (RCTs) being regarded as the most credible form of evidence (Gupta et al., 2016; Smith, 2014). EBM recognizes that clinical experience and patient values should be considered when deciding which intervention to implement, but these are often seen as supplementary to research evidence. While RCTs and other research methodologies are important components of the evidence base, we must recognize the equity-related limitations of these components. Many studies are not conducted with or for diverse (with respect to race, ethnicity, gender, and other aspects of identity) or systemically oppressed populations. Some such as the infamous Tuskegee Syphilis Study have also used exploitative methods (Martinez et al., 2010; Lamar Soutter Library, 2020; Brandt 1978).

Although MCH (and public health generally) is a separate discipline from medicine, many of its philosophies, principles, and theories are grounded in EBM; this extends to what we consider evidence and how it is valued (Brownson et al., 2009). Evidence-based public health builds on the principles of EBM by increasing the role of community needs and preferences in informing decisions. Entities such as the Centers for Disease Control and Prevention (CDC) have recognized that in addition to the “best available research evidence,” i.e., those derived from systematic research methodologies, other forms of evidence are important when making decisions (Puddy & Wilkins, 2011). These are:

- **Experiential evidence**, which is derived from professional insight, understanding, skill, and expertise accumulated over time; and.
- **Contextual evidence**, which is derived from actors that address whether a strategy is useful, feasible to implement, and accepted by a particular community.

Despite this acknowledgment, the CDC does not provide robust guidance on how to use these types of evidence for decision making across professional functions, e.g., practice planning, implementation, and evaluation (CDC, n.d.-a, CDC, n.d.-b). This is in stark contrast to the explicit guidance it provides in its *Understanding Evidence* report regarding the types of experimental research designs that are the strongest and most effective (Puddy & Wilkins, 2011). The guidance gap is a subtle but important indicator of a difference in investment – not just in funding for efforts that capture and attempt to elevate experiential and contextual evidence, but in training the current and preprofessional workforce to both engage in and systemically embed those practices. It inadvertently devalues the experiences and perspectives of the people and communities most directly impacted by MCH and other public health initiatives. As one of the leading public health authorities, how the CDC frames evidence has a deep impact on the ways in which our field prioritizes and approaches learning – from whom and how should we be gathering information?

## The Main Point

### Why does it Matter how we define evidence?

Evidence is central to all MCH decision-making processes, continuously interacting with and influencing our work. The percent of Health Resources and Services Administration notices of funding opportunities including the term “evidence-based” has increased from 5.4% to 2008 to 48.8% in 2021 (Grants.gov, n.d.). This increase emphasizes the danger of operating with a narrow view of evidence focused solely on research; entities with the resources to conduct experimental studies and to submit to peer-reviewed journals may be given priority over community-based organizations (CBOs) for funding opportunities as they may be viewed as more credible approaches and better able to generate valuable evidence. This dynamic leads to a cycle of exclusion where community-rooted practices are diminished within or kept out of the evidence canon considered actionable for public health decision making because they do not appear in scholarly literature. Instead, CBOs are often competing for limited pools of community-oriented funding or relegated to supplemental roles (e.g., advisory, consultative) on proposals led by government agencies, universities, or private firms.

Breaking this cycle of exclusion requires shifting the MCH field’s evidence paradigm, establishing and consistently reinforcing the importance of contextual and experiential evidence in determining the efficacy of a practice. These types of evidence are not just accessories to research evidence, but integral components of all evidence-based decision making and critical types of evidence in their own right.

### Shifting the evidence paradigm

When determining the scope of a challenge and making decisions about how to address them, contextual and experiential evidence, alongside research evidence, should be utilized for two distinct purposes: (1) to support the effectiveness of a given practice generally, and (2) to support that the practice will work in specific contexts. Community-defined evidence (CDE), a “set of practices that communities have used and found to yield positive results as determined by community consensus over time,” is one example of contextual evidence being used to fulfill this first purpose (Martinez, 2008; Martinez et al., 2010). These practices may or may not have been measured empirically but have reached a level of acceptance within a geographically- or identity-defined community. CDE takes into consideration a population’s worldview as well as historical and social contexts acknowledging that communities are experts about what “works for them” (Martinez, 2008). Examples of prioritizing CDE in decision making could look like allocating funds to a neighborhood resource center to scale up an existing adolescent health initiative that is widely regarded by community members as being instrumental in supporting youth well-being, ensuring that community members are provided with adequate resources and support to identify examples of CDE at the local level, and supporting a local health education organization to design and implement an evaluation of an engagement technique that incorporates art and storytelling into a sexual health curriculum. Capturing the insights, understanding, skills, and expertise of people with lived experience (PWLE) through qualitative methods such as focus groups, surveys, and key informant interviews (i.e., experiential evidence) can also provide nuanced information about the impact and effectiveness of a practice. Research approaches such as community-based participatory research (CBPR), a “collaborative approach to research that equitably involves, for example, community members, organizational representatives, and researchers in all aspects of the research process,” are yet another form of evidence that can support the effectiveness of a practice while amplifying the voices of those impacted by the practice (Israel et al., 1998).

Shifting what and how our field considers evidence will require the intentional co-creation of evidence with communities and people with lived experience (PWLE) through methods that center their voices and opinions. Even if a practice has strong research, contextual, and experiential evidence supporting its impact generally, it must also be perceived by intended beneficiaries as being in alignment with their values and life circumstances. Efforts to decolonize research and CBPR are examples of the transformative impact of using methodology to center community voices. Contextual and experiential evidence gathered in partnership with community representatives should be regarded as key components of assessing fit. Of note, uplifting contextual and experiential evidence in no way suggests that research evidence is not of critical importance when making evidence-based decisions. Rather, doing so aims to rebalance the currently accepted evidence equation that unintentionally contributes to inequitable population-level MCH outcomes.

### The role of title V

As is often raised in the context of racial equity work, truly shifting power (Farhang & Gaydos, 2021) involves those benefiting from current practices ceding that power to those that have been systemically blocked from leading (and in most cases simply participating in) these practices. It is only then that we can expect changes in who really benefits from using evidence to inform decisions and the resulting practices. MCH professionals are well-positioned to lead this power shift. The Title V Maternal and Child Health (MCH) Services Block Grant to States Program “envisions a nation where all mothers, infants, children aged 1 through 21 years, including children with special health care needs, and their families are healthy and thriving” (Maternal and Child Health Bureau [MCHB], 2020). This vision isn’t singular, but rather suggests that what we do must resonate with (i.e., acknowledge intersecting identities and collections of circumstances) the people for whom our work is intended to benefit. We have the platform to influence the evidence narrative about what constitutes being healthy and thriving.

The Guidance and Forms for the Title V Application/Annual Report, Ninth Edition (2020) encourages jurisdictions to “collaborate with community leaders/organizations and families of every background in needs/assets assessments, program planning, service delivery and valuation/monitoring/quality improvement activities.” This statement rightfully calls out the importance of seeking and embedding contextual and experiential evidence across processes – not just in assessing needs – necessitating a foundational set of skills that the Maternal and Child Health Leadership Competencies: Version 4.0 (MCHB, 2018) touches on in areas such as ethics, cultural competency, family-professional partnerships, and working with communities and systems.

### Workforce call to action

So how do we build the necessary pressure to shift the evidence paradigm and fully recognize the value of contextual and experiential evidence? As individuals, shifting power requires the cultivation of sustained momentum and enthusiasm, as well as training (inclusive of academic preparation and continuing professional education) to develop strategic and reflective skills in three inextricably linked domains:

- **Communication**: Forging a holistic view of evidence demands active listening and establishing multiple channels of open and transparent communication with impacted community members. This influences the degree to which we as MCH professionals can capably and authentically present community assets, resources, and priorities.
- **Equitable leadership**: We must build awareness of programs and policies (past and present) that may enable or hinder community partnerships. A justice orientation is essential across the lifecycle of evidence assessment, creation, implementation, and dissemination. It also necessitates the establishment of opportunities for community members to access training and mentorship that builds on their existing expertise and augments their capacity to critically assess and produce evidence.
- **Change management**: We acknowledge that MCH professionals may need to adhere to traditionally accepted frameworks for research practice due to funding requirements, leadership expectations, politics, and time and resource constraints. Working around these constraints requires a commitment to shifting power through change management approaches, including targeted communication with funders and other influential partners about the benefits of valuing contextual and experiential evidence and promoting narratives that affirm community capacity.

The stark, persistent racial and other identity- and circumstance-based disparities in health outcomes across domains mean we must work urgently to break cycles of community exclusion in evidence generation; this begins with intentionally emphasizing the importance of contextual and experiential evidence when making decisions about which practices we implement and replicate. There will be necessary stumbling and tensions between funders, researchers, and PWLE as we rebalance and change longstanding norms. This tension presents exciting opportunities for MCH to better resemble the anti-oppressive field we collectively envision, and to model this for the rest of public health. Together, Title V can help the MCH field shift the evidence paradigm by:

- Developing and investing in partnerships with community-rooted organizations that includes holistic evaluation of their practices;
- Thinking critically about what evidence currently guides our work to identify gaps in the types of evidence we are not adequately considering, and identifying with whom we need to connect to gather that information;
- Talking with existing and potential funders about raising the profile of contextual and experiential evidence within their mission statements, funding proposals, and scoring rubrics; and.
- Creating and sharing outcome measures that rely primarily on contextual and experiential evidence, which can contribute to broader community accountability efforts.

The MCH workforce, inclusive of all levels of leadership, can and should position the communities we’re responsible for serving to set their own priorities and define, develop, and disseminate evidence that is representative of their priorities. Failing to do so will increase the distance between the intention and impact of MCH practices, result in community expertise being ignored, and allow disparate MCH outcomes to persist. Uplifting evidence that centers the voices of communities and PWLE can contribute to power and infrastructure building, as well as the cultivation of mutually beneficial relationships between community members and Title V programs. Evidence co-creation is an essential pathway to building and sustaining trust, and its role in strengthening MCH efforts to advance racial and other forms of equity cannot be overstated.

## Data Availability

Not applicable.

## Code Availability

Not appliable.
